# Red Blood Cell-Associated Features of Adenoviral Vector-Linked Venous Thrombosis

**DOI:** 10.3390/ijms262311606

**Published:** 2025-11-29

**Authors:** Hanjin Park, Ok-Nam Bae, Sungbin Choi, Eunha Lee, Jun Chang, Han Young Chung

**Affiliations:** 1 College of Pharmacy, Institute of Pharmaceutical Science and Technology, Hanyang University, Ansan 15588, Republic of Korea; opensky95@hanyang.ac.kr (H.P.); onbae@hanyang.ac.kr (O.-N.B.); hjklkl123@hanyang.ac.kr (S.C.); 2 College of Pharmacy, Chungnam National University, Daejeon 34134, Republic of Korea; milkway1511@cnu.ac.kr; 3 Graduate School of Pharmaceutical Sciences, Ewha Womans University, Seoul 03760, Republic of Korea; tcell@ewha.ac.kr

**Keywords:** adenoviral vector vaccines, adenovirus type 5 (Ad5), red blood cells, venous thrombosis, RBC shape changes, thrombin generation

## Abstract

Adenoviral vector vaccines were pivotal for COVID-19 control, but postmarketing safety surveillance has identified venous-predominant thrombotic risks not fully explained by platelet-centric mechanisms. We tested an RBC-associated hypothesis using an Ad5 vector-rAd/HA(PR8) rat model within a predefined sub-hemolytic window (<10% hemolysis). Ex vivo, we quantified RBC surface phosphatidylserine (PS) exposure, morphology remodeling by scanning electron microscopy, and microvesicle generation, all aligning with increased procoagulant activity. RBCs also exhibited dose-dependent increases in thrombin generation 4 h after intravenous exposure (10^8^–10^9^ OPU/Rat). In vivo, an inferior vena cava thrombosis model showed a pronounced, dose-responsive rise in thrombus burden, consistent with increased thrombogenic potential. Together, these integrated data provide experimental evidence consistent with RBC involvement under adenoviral exposure, supporting a biologically plausible link to the venous-predominant epidemiology observed during the COVID-19 vaccination era. Reported clinical adenoviral vaccine doses are of the same order of magnitude as the exposures tested here, supporting translational relevance while not implying inter-species or product equivalence. Incorporating RBC-focused endpoints, including PS exposure, morphology indices, microvesicle counts, and thrombin generation, into preclinical and early clinical assessments may enhance safety evaluation and inform vector design to mitigate venous thrombotic risk.

## 1. Introduction

Adenoviral vector-based vaccines have been pivotal in global efforts to control the COVID-19 pandemic due to their rapid development and widespread distribution [1,2]. Despite their overall efficacy, these vaccines, notably AstraZeneca’s ChAdOx1 nCoV-19 (AZD1222) and Johnson & Johnson’s Ad26.COV2.S, have been associated with rare but serious thrombotic complications such as vaccine-induced immune thrombotic thrombocytopenia (VITT) [3,4]. Recent comparative pharmacoepidemiologic analyses indicate higher risks of venous thrombotic events, particularly cerebral venous thrombosis (CVT), following adenoviral vector vaccines compared with mRNA platforms [5,6]. For example, a Nordic self-controlled case series reported a rate ratio of 12.04 (95% CI, 5.37 to 26.99) for CVT after AZD1222, underscoring a venous-predominant signal not fully explained by platelet-centric mechanisms alone [6]. Although the clearest post-authorization signals were observed with ChAdOx1 (chimpanzee adenoviral vector) and Ad26.COV2.S (human adenovirus 26), multiple adenoviral vectors display blood-interacting capsid features at the host interface, supporting the idea that platform-level mechanisms may extend beyond any single product [7,8,9]. Accordingly, there is a need to delineate non-platelet contributors that could preferentially influence venous thrombus formation under adenoviral exposure.

At the same time, adenoviral COVID-19 vaccines have provided substantial public-health benefits. In pooled phase 2/3 trials, the ChAdOx1 nCoV-19 (AZD1222) vaccine significantly reduced symptomatic COVID-19 and severe disease, contributing to control of acute SARS-CoV-2 infection [10]. Moreover, observational data indicate that COVID-19 vaccination is associated with a decreased likelihood of long COVID symptoms [11,12] and a substantial reduction in referrals to dedicated long COVID clinics [13], suggesting that vaccination also mitigates post-acute sequelae. Within this broader benefit–risk context, our study focuses on a rare, venous-predominant thrombotic signal and aims to clarify RBC-linked mechanisms rather than to question the overall value of adenoviral vaccination.

Conventionally, platelet activation is primarily associated with arterial thrombosis, whereas red blood cells (RBCs) are increasingly recognized to play critical roles in venous thrombosis [14,15]. While platelet activation and the production of anti-PF4 antibodies have been considered primary mechanisms of vaccine-associated thrombosis, accumulating evidence indicates that additional cellular elements contribute in venous settings [16]. Recent insights characterize RBCs as active participants rather than passive elements within coagulation processes [17,18]. In low-shear venous beds, the abundance and morphology of RBCs materially shape clot architecture and stability [19,20]. Within this context, adenoviral exposure may potentiate venous thrombogenesis through RBC-associated processes, including provision of procoagulant surfaces and modulation of thrombin generation [21,22].

Emerging clinical and translational observations have documented alterations in RBC properties, including deformability and morphology, in COVID-19-related and thrombotic contexts [23,24]. These RBC changes provide a biologically plausible basis for potential involvement in vaccine-associated thrombosis, particularly when adenoviral vector exposure is relevant. Among human adenoviruses, adenovirus type 5 (Ad5) is widely used across vaccine and gene-delivery platforms and is well characterized for direct interactions with circulating blood cells, including sequestration by RBCs [7,21]. Given this tractability, Ad5-based systems permit controlled testing of adenovirus–RBC interactions as a model for class-level blood interfaces [25]. Taken together, these observations motivate direct experimental tests to determine whether adenoviral vectors can elicit RBC-associated prothrombotic responses that align with venous risk signals. To maintain translational relevance without overinterpretation, we also selected exposure levels within the same order of magnitude as clinically used adenoviral vaccine doses [26,27], framing our work as a mechanism-oriented bridge rather than a product-specific replication of post-authorization findings.

However, direct experimental evidence explicitly linking adenoviral vectors to RBC-mediated thrombosis remains limited. Given the venous predominance of post-licensure signals and the recognized influence of RBCs on thrombosis under low-shear conditions [5,15], we designed our study to focus on venous-context, blood-interface endpoints within a controlled rat system, specifically RBC shape changes in circulation and whole-blood thrombin-generation readouts. Using an Ad5-based rAd/HA(PR8) model, we first characterized ex vivo RBC shape changes by electron microscopy, second quantified ex vivo thrombin generation, and third assessed in vivo venous thrombus formation, to generate data connecting adenoviral exposure with venous thrombogenicity involving RBCs. This Ad5-focused design probes platform-relevant biology at the blood interface and is intended to complement, not substitute for, platelet-centered explanations of vaccine-associated thrombosis observed with other adenoviral products [7,9]. In line with this scope, we evaluate whether integrated readouts across scales (RBC morphology, thrombin generation, and venous thrombosis in vivo) are consistent with RBC involvement under intravenous exposure, without inferring product equivalence across serotypes or vaccines. Consistent with venous physiology, we prioritize RBC-oriented endpoints alongside platelet-centric measures. Finally, we outline the rationale for incorporating RBC-focused measurements as hypothesis-driven exploratory endpoints for preclinical screening, with implications for clinical risk assessment.

## 2. Results

### 2.1. Vector Overview, Sub-Hemolytic Window, and Study Workflow

**Figure 1:**
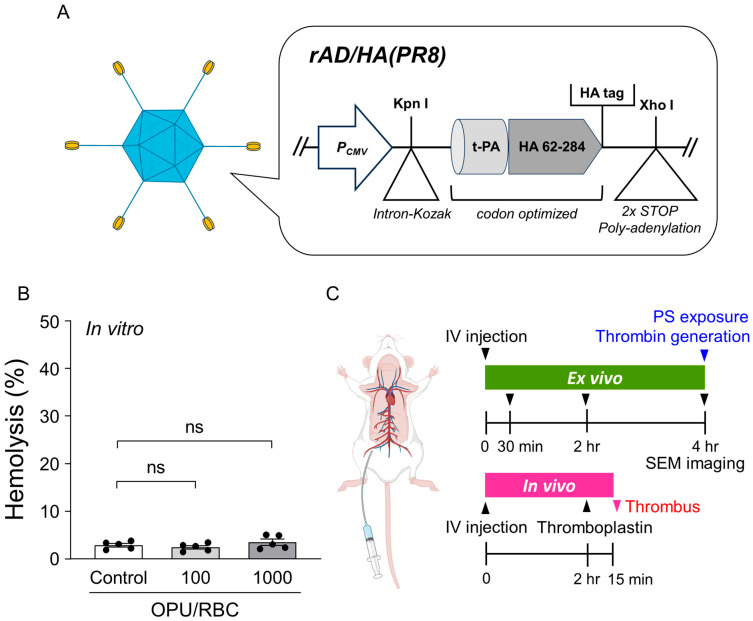
Experimental design within a sub-hemolytic range for Ad5 vector-rAd/HA(PR8). (**A**) Schematic illustration of Ad5 vector-rAd/HA(PR8), encoding the hemagglutinin (HA1) domain (amino acids 62–284) from influenza A/Puerto Rico/8/34 with a tissue plasminogen activator (tPA) signal peptide for secretion enhancement. (**B**) In vitro hemolysis of rat RBCs across indicated particle-to-cell ratios (OPU/RBC), confirming a sub-hemolytic exposure window (<10% total lysis) measured at 4 h. Data represent mean ± SEM (*n* = 5). Statistical significance was determined by one-way ANOVA followed by Dunnett’s post hoc test versus control. ns, not significant. (**C**) Overview of experimental approaches used in this study: ex vivo assays (**top panel**) evaluating RBC phosphatidylserine (PS) exposure, thrombin generation, and RBC shape changes at multiple intervals, and in vivo validation (**bottom panel**) employing a rat venous thrombosis model.

The adenovirus vector used in this study, Ad5 vector-rAd/HA(PR8), was designed to express the soluble globular head domain (amino acids 62–284) of the influenza hemagglutinin (HA) protein from influenza A/Puerto Rico/8/34 (H1N1), coupled with a human tissue plasminogen activator (tPA) signal peptide for enhanced secretion (Figure 1A). To probe early RBC functional and structural responses while minimizing overt lysis, we predefined a sub-hemolytic window as <10% total hemolysis and optimized exposure parameters accordingly. All hemolysis assays in this study were performed using rat RBCs. Across the tested particle-to-cell ratios (0–1000 OPU/RBC) and time points at 37 °C with gentle shaking, RBC hemolysis remained within this sub-threshold range, confirming that the subsequent readouts were obtained under a sub-hemolytic exposure window (<10% total lysis) in which overt membrane rupture was minimized (Figure 1B). Figure 1C summarizes the study workflow. Following intravenous (IV) injection, ex vivo assessments were performed at predefined time points (e.g., 0.5 h, 2 h, 4 h) to analyze SEM-based RBC shape changes and quantify thrombin generation. In vivo venous thrombosis was evaluated under thromboplastin-triggered conditions, using the same vector doses that satisfied the sub-hemolytic criterion in ex vivo testing, ensuring that hemolysis did not confound downstream analyses. These settings allowed us to assess procoagulant activity and morphology under conditions in which bulk hemolysis was minimized.

### 2.2. Ex Vivo Evaluation of PS Exposure, RBC Morphology, and MV Generation in Relation to Thrombin Generation

**Figure 2:**
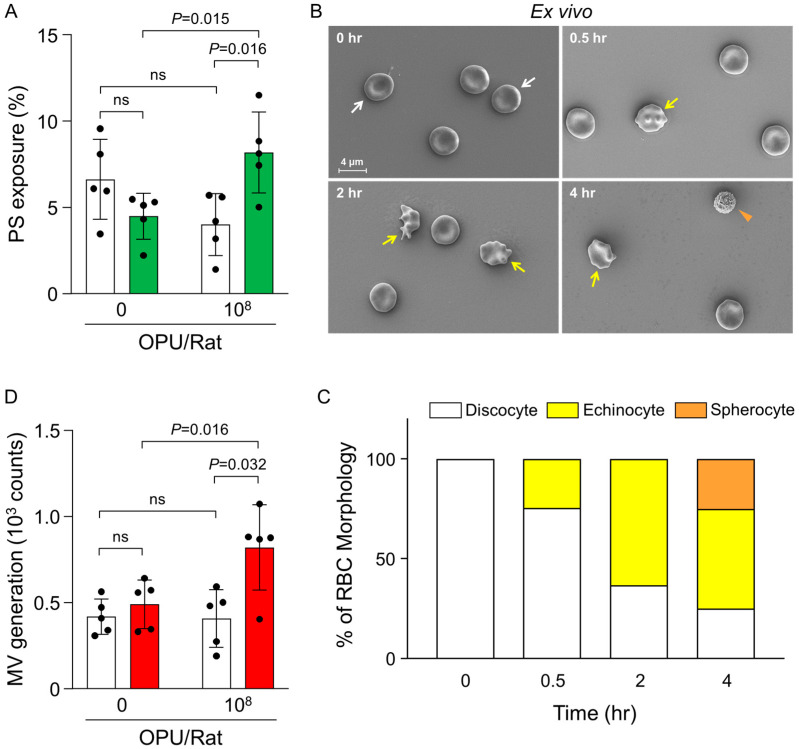
Ex vivo assessment of PS exposure, RBC morphology, and microvesicle generation after intravenous administration of Ad5 vector-rAd/HA(PR8). (**A**) RBC surface phosphatidylserine (PS) exposure measured by annexin V binding in blood collected from rats receiving vehicle (0) or Ad5 vector-rAd/HA(PR8) (10^8^ OPU/Rat) at baseline and 4 h after injection. White bars indicate baseline (0 h), and green bars indicate 4 h after injection. Data are presented as mean ± SEM (*n* = 5). Statistical significance was determined by one-way ANOVA followed by Dunnett’s post hoc test. (**B**) Representative SEM images of rat RBCs collected at 0, 0.5 h, 2 h, and 4 h after intravenous administration of Ad5 vector-rAd/HA(PR8) (10^9^ OPU/rat). White arrows indicate discocytes, yellow arrows indicate echinocytes, and the orange arrowhead denotes a spherocyte. Scale bar, 4 μm. (**C**) Quantification of RBC morphologies from ex vivo SEM images after intravenous administration (10^9^ OPU/rat), expressed as the percentage of total RBCs by morphology (discocyte, echinocyte, spherocyte) at the indicated time points (*n* = 5). Stacked bars depict relative proportions. (**D**) RBC-derived microvesicle (MV) generation in blood collected from rats receiving vehicle (0) or Ad5 vector-rAd/HA(PR8) (10^8^ OPU/Rat) at baseline and 4 h after injection, quantified by flow cytometry. White bars indicate baseline (0 h), and red bars indicate 4 h after injection. Data are presented as mean ± SEM (*n* = 5). Statistical significance was determined by unpaired two-tailed *t*-test. ns, not significant.

Under the predefined sub-hemolytic conditions, we first evaluated phosphatidylserine (PS) exposure on RBCs as a marker of procoagulant activity [28,29]. Four hours after intravenous administration of Ad5 vector-rAd/HA(PR8) (10^8^ OPU/Rat), the percentage of PS-positive RBCs was significantly increased compared with vehicle (*p* = 0.016; Figure 2A). Because externalized PS provides a catalytic surface for assembly of the factor Xa/Va complex [30,31], these data indicate that adenoviral exposure enhances the procoagulant potential of circulating RBCs.

We next examined ex vivo RBC morphology over time following vector administration. In Figure 2B, white arrows indicate discocytes (biconcave cells), yellow arrows denote echinocytes (cells bearing short, regularly spaced spicules), and the orange arrowhead marks a representative spherocyte (near-spherical cell with loss of central pallor). Scanning electron microscopy (SEM) images showed that at 0.5 h RBCs were largely discocytic with occasional early echinocytic features; by 2 h, echinocytes became prominent; and by 4 h, spherocytes appeared alongside sustained echinocytosis, consistent with progressive remodeling under continued vector exposure. Quantitative analysis confirmed a redistribution from discocytes toward echinocytes and, at later time points, spherocytes (Figure 2C).

To link these morphological transitions to vesiculation, we quantified RBC-derived microvesicles (MVs) 4 h after vector administration. MV counts were significantly higher in rats receiving 10^8^ OPU/Rat than in vehicle-treated controls (Figure 2D). Given that MVs are shed from remodeled RBCs and are enriched in PS, the increase in MV generation during the transition from echinocytes to spherocytes supports a close coupling between RBC shape change, vesicle release, and provision of additional procoagulant surface.

**Figure 3:**
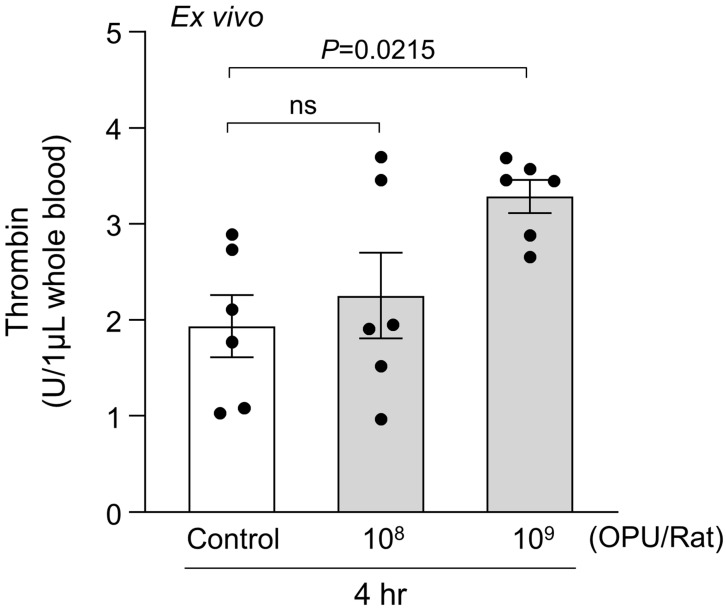
Dose-dependent ex vivo thrombin generation after intravenous Ad5 vector-rAd/HA(PR8) administration. Whole-blood thrombin generation measured 4 h post-dose in rats receiving 10^8^ or 10^9^ OPU/Rat versus vehicle control. Units are U per 1 μL whole blood. Each dot represents an individual rat. Data represent mean ± SEM (*n* = 6). Statistical significance was determined by one-way ANOVA followed by Dunnett’s post hoc test versus control. ns, not significant.

Finally, we assessed thrombin generation in whole blood 4 h after dosing with 10^8^ or 10^9^ OPU/Rat. Using RBCs isolated from blood drawn from the inferior vena cava, thrombin activity showed a dose-responsive increase, with 10^9^ OPU/Rat exceeding 10^8^ OPU/Rat and vehicle and achieving statistical significance versus control (*p* = 0.0215; Figure 3). Taken together, the increased PS exposure, enhanced MV generation accompanying echinocyte-to-spherocyte transitions, and dose-dependent thrombin generation indicate an RBC-associated procoagulant response under sub-hemolytic conditions in this rat model.

### 2.3. In Vivo Assessment of Venous Thrombosis Under a Sub-Hemolytic Window

**Figure 4:**
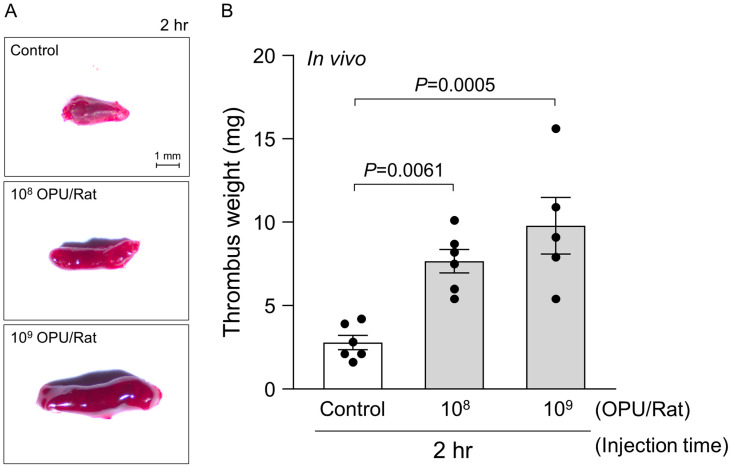
In vivo validation of RBC-mediated thrombosis following intravenous administration of Ad5 vector-rAd/HA(PR8). (**A**) Representative images of thrombi formed within the rat inferior vena cava (IVC) 2 h after intravenous injection of Ad5 vector-rAd/HA(PR8) at indicated doses (10^8^ and 10^9^ OPU/Rat) or vehicle control. Scale bar, 1 mm. (**B**) Quantitative analysis demonstrating a significant dose-dependent increase in thrombus weight (mg) induced by Ad5 vector-rAd/HA(PR8). Data represent mean ± SEM (*n* = 5–6). Statistical significance was determined by one-way ANOVA followed by Dunnett’s post hoc test versus control.

To extend these findings to a whole-blood context, we evaluated inferior vena cava thrombosis in rats 2 h after intravenous (IV) administration of Ad5 vector-rAd/HA(PR8) (10^8^ or 10^9^ OPU per rat) followed by standardized thromboplastin triggering (Figure 4). Representative thrombus images (Figure 4A) show a visible increase in clot size with dose, consistent with vector-linked enhancement of venous thrombogenesis. Quantification of thrombus weight (*n* = 5–6) confirmed that both 10^8^ and 10^9^ OPU per rat exceeded vehicle control at the 2 h endpoint (*p* = 0.0061 and *p* = 0.0005 vs. control, respectively; Figure 4B). Together with ex vivo data showing SEM-defined remodeling from discocytes to echinocytes and spherocytes, and dose-dependent thrombin generation (Figure 2 and Figure 3), these in vivo results support an RBC-associated pattern in which adenoviral exposure promotes venous thrombogenesis under a sub-hemolytic window, in parallel with structural remodeling and heightened procoagulant activity.

## 3. Discussion

**Figure 5:**
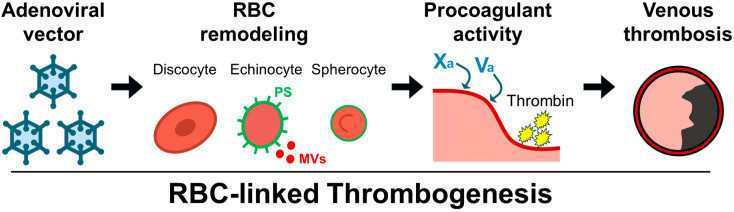
Schematic of an RBC-linked pathway for adenoviral vector-associated venous thrombogenesis. Following intravenous administration of Ad5 vector-rAd/HA(PR8), red blood cells remodel from discocytes to echinocytes to spherocytes. During this remodeling, green outlines denote increased surface phosphatidylserine (PS) exposure and red dots represent PS-rich microvesicles (MVs), together contributing to morphology-dependent procoagulant activity that supports factor Xa/Va-dependent thrombin generation and culminates in venous thrombosis in the rat IVC model. The diagram is not to scale and serves as a conceptual overview integrating the ex vivo and in vivo findings.

Building on the pandemic-era experience where vaccine-associated thrombosis was interpreted largely through a platelet-centric view, our results support an RBC-aware framework that is particularly relevant to venous beds [22]. Within this framework, RBCs are positioned as active contributors to venous thrombogenesis under adenoviral vector exposure, complementing rather than replacing the platelet/PF4 paradigm [9,32]. Mechanistically, remodeling of RBC populations can be linked to enhanced thrombin generation in whole blood, providing a functional bridge between structural changes and coagulation output [18,22,33]. These findings align with venous-predominant epidemiology after adenoviral vaccination, supported by pharmacovigilance and post-authorization analyses [5,6], especially in low-shear venous environments where RBC abundance, rheology, and shape transitions influence clot architecture and stability [14,20]. A schematic overview is shown in Figure 5, illustrating an RBC-linked sequence in which adenoviral exposure drives RBC remodeling from discocytes, through echinocytes, to spherocytes, accompanied by increased surface PS exposure and shedding of PS-rich microvesicles, which together support morphology-dependent thrombin generation and culminate in venous thrombosis.

An important methodological point is the route of administration. In our study, the adenoviral vector was given intravenously, whereas currently used adenoviral COVID-19 vaccines are injected intramuscularly (IM) [34,35]. We chose the intravenous route deliberately to create a situation where the vector is immediately and evenly exposed to blood cells, so that we could clearly examine how it interacts with RBCs and promotes procoagulant activity. In real-world vaccination, intramuscular injection is expected to produce lower and more gradual entry of the vector into the bloodstream [36,37]. In that setting, vector particles may reach the circulation at low levels after IM injection, either via drainage from the injection site into the vascular system or, rarely, via accidental intravascular injection. Therefore, our results should be understood as showing what circulating adenoviral vectors are capable of doing to RBC-linked procoagulant pathways under a high blood-exposure scenario, rather than as a direct reproduction of routine vaccination practice or a precise estimate of thrombotic risk at clinical doses and routes.

To avoid overinterpretation and remain aligned with measured endpoints, we focused strictly on three prespecified readouts, namely (i) SEM-defined RBC remodeling from discocytes to echinocytes and spherocytes, (ii) increased ex vivo thrombin generation, and (iii) greater in vivo venous thrombus burden after vector exposure. All experiments were conducted within a predefined sub-hemolytic window (<10% total hemolysis), which minimizes overt hemolysis. Although we cannot entirely exclude minor effects of low-level lysis, the concordant changes in PS exposure, MV generation, and RBC shape remodeling indicate that the observed prothrombotic signal is driven predominantly by RBC remodeling-related mechanisms. Because venous beds provide a low-shear, RBC-dense milieu [14,38], our choice of the inferior vena cava (IVC) thrombosis model situates the in vivo endpoint in a context where RBC contributions are expected to be prominent. We observed dose-dependent enhancement of thrombin generation following exposure to Ad5 vector-rAd/HA(PR8), consistent with evidence that circulating blood cells, including RBCs, contribute substantially to thrombin generation in whole blood [39,40]. Additionally, we observed progressive RBC shape transitions from echinocytes to spherocytes, a pattern previously linked to procoagulant states and to remodeling of clot architecture [41]. Importantly, these structural and functional readouts coincided with a 2 h increase in IVC thrombus burden without invoking unmeasured molecular intermediates. The different sampling times (ex vivo 4 h versus in vivo 2 h) were prespecified by study design and do not imply biological inconsistency. Another important limitation of our work is the relatively short observation window. All ex vivo measurements were performed within 0–4 h after vector administration, and in vivo venous thrombosis was assessed 2 h after dosing. These time points were chosen to capture the acute phase of vector–blood interactions, including early RBC remodeling, PS exposure, and MV shedding under sub-hemolytic conditions. By contrast, clinical vaccine-associated thrombotic events typically emerge over several days rather than hours [42,43]. Our data therefore describe the immediate capacity of circulating adenoviral vectors to induce RBC-linked procoagulant changes, but they do not establish whether these changes are transient, reversible, or sustained over longer periods. Practical and ethical constraints, including the terminal design of the rat IVC thrombosis model and the requirement for freshly collected blood for ex vivo assays, precluded systematic assessment at later time points in the present study. Future investigations incorporating extended time courses and models that recapitulate the delayed immune and inflammatory phases after vaccination will be needed to link these early mechanistic findings more directly to clinical timelines. Despite these limitations, the aligned increases in thrombin generation, venous thrombus burden, and RBC shape remodeling support an RBC-associated contribution to adenoviral vector-linked venous thrombogenesis.

At the population level, adenoviral vector vaccination is associated with a venous-predominant signal (notably CVT) compared with mRNA platforms [44,45]. Our controlled model provides a coherent bridge to this epidemiology by demonstrating concordant sub-hemolytic RBC remodeling, increased ex vivo thrombin generation, and higher IVC thrombus burden. While the animal model does not recapitulate the full clinical spectrum, these aligned signals support using RBC-based markers as informative complements to platelet-centric assessments in adenoviral platform safety evaluation [46,47].

The adenoviral vector doses used in our study, 10^8^ and 10^9^ OPU per rat, were selected based on their ability to induce measurable biological effects in animal models. Translating these doses to human vaccination scenarios requires careful consideration. In addition, the Ad5-based vector used here (rAd/HA(PR8)) is distinct from the ChAdOx1 nCoV-19 and Ad26.COV2.S vectors that have been epidemiologically linked to clinical VITT, and different adenoviral serotypes exhibit capsid- and platform-specific patterns of interaction with blood factors such as PF4 and other plasma proteins [9,48,49]. For this reason, our findings should be viewed as hypothesis-generating evidence for RBC-associated mechanisms at the level of adenoviral vector–blood interfaces, rather than as direct evidence about the risk profile or behavior of any particular licensed vaccine product. Clinically administered adenoviral vaccines typically use doses on the order of 10^10^ to 10^11^ viral particles per person, aligning in magnitude with the exposures tested here [50,51]. Given these similarities in dosing scale, our results may be informative for human clinical contexts, while still requiring caution in extrapolation across species and vaccine platforms. While we do not imply direct inter-species equivalence, the order-of-magnitude concordance supports translational plausibility that comparable RBC-associated pathways may contribute in clinical settings. Practically, RBC-focused readouts, including thrombin generation and morphology indices under sub-hemolytic conditions, could be integrated as adjuncts to platelet-centric endpoints in preclinical and early clinical assessments.

In summary, by anchoring interpretation to measured phenomena (RBC remodeling, ex vivo thrombin generation, and in vivo venous thrombosis) and by explicitly operating within a sub-hemolytic window, this work sharpens an RBC-aware perspective on adenoviral vector-associated thrombosis that is conceptually consistent with venous-predominant epidemiology [44]. This paradigm sharpening does not dispute platelet involvement; rather, it broadens the evaluative lens to include RBC-linked contributions in low-shear venous contexts.

## 4. Materials and Methods

### 4.1. Adenovirus Vector Preparation

The replication-defective recombinant adenovirus serotype 5 vector (Ad5 vector-rAd/HA(PR8)), expressing the soluble globular head domain of the hemagglutinin (HA) protein from influenza A/Puerto Rico/8/34 (H1N1), was generated following the method described previously [25]. Briefly, a synthetic DNA sequence encoding the HA1 domain (amino acid residues 62–284 of PR8) was synthesized and fused with an HA-tag (TOP Gene Technologies, Montreal, QC, Canada). After sequence verification, the synthesized DNA was subcloned into the pGEM-T Easy vector (Promega, Madison, WI, USA). Subsequently, the HA1 coding region was amplified, and a signal peptide derived from human tissue plasminogen activator (tPA) was incorporated at the N-terminus to enhance secretion. The complete open reading frame was then excised and cloned into the pShuttle-CMV vector. Recombinant adenoviruses were generated via homologous recombination and purified using established protocols [25]. The viral stock was stored at −80 °C in stabilizing buffer consisting of 10 mM Tris (pH 7.4), 150 mM NaCl, 6% sucrose, 0.04% Tween-80, 200 µM EDTA, and 1% ethanol until use.

### 4.2. Ethical Approval and Animal Preparation

All animal experiments were approved by the Ethics Committee of the Animal Service Center at Hanyang University (Approval No.: IACUC 2025-0061A). Male Sprague Dawley (SD) rats (280–350 g) were anesthetized with urethane for blood collection and in vivo thrombosis experiments.

### 4.3. In Vitro Measurement of Hemolytic Activity

Rat red blood cells (RBCs) were collected, washed three times with PBS (pH 7.4), and adjusted to 5 × 10^7^ cells/mL. RBC suspensions were incubated with the Ad5-based rAd/HA(PR8) vector at defined particle-to-cell ratios (e.g., 0–1000 OPU per RBC) at 37 °C with gentle shaking (1000 rpm) for the indicated time points. After 4 h incubation, samples were centrifuged at 10,000× *g* for 2 min, and hemoglobin released into the supernatants was measured at 540 nm using a spectrophotometer. Complete lysis induced by 1% Triton X-100 was used as the 100% reference, and PBS-treated cells served as the 0% control.

### 4.4. Ex Vivo Assessment of Thrombin Generation

Ex vivo thrombin generation was assessed using whole blood obtained from rats intravenously injected with Ad5 vector-rAd/HA(PR8) at doses of 10^8^ or 10^9^ OPU/rat. Four hours post-injection, blood samples were collected via the abdominal aorta into citrate-dextrose solution and immediately processed. Following centrifugation at 200× *g* for 15 min, isolated RBCs were washed twice with phosphate-buffered saline (PBS; pH 7.4) and resuspended to a density of 5 × 10^7^ cells/mL in Ringer’s solution (125 mM NaCl, 5 mM KCl, 1 mM MgSO_4_, 32 mM HEPES, 5 mM glucose; pH 7.4).

For thrombin generation assays, RBC suspensions were incubated with factor Xa (5 nM) and factor Va (10 nM) in Tyrode’s buffer containing 2 mM CaCl_2_ at 37 °C for 3 min. Subsequently, human prothrombin (2 µM) was added, and incubation continued for an additional 3 min. The reaction was terminated using EDTA-containing buffer, and thrombin activity was quantified spectrophotometrically at 405 nm. Thrombin activity (U) was defined as 1 µmol of chromogenic product formed per minute, and ex vivo values were expressed as U per µL of whole blood.

### 4.5. Ex Vivo Assessment of Phosphatidylserine Exposure by Flow Cytometry

For ex vivo assessment of red blood cell (RBC) surface phosphatidylserine (PS), rats were intravenously injected with Ad5 vector-rAd/HA(PR8) at doses of 0 or 10^8^ OPU/rat. Four hours after injection, whole blood was collected via the abdominal aorta into citrate-dextrose solution and processed immediately. Then we isolated rat RBCs from the blood samples after centrifugation and 3 washes and stained the PS on the rat RBC membrane with annexin-V FITC (BD Bioscience, Franklin Lakes, NJ, USA) and PE Rat Anti-Mouse TER-119/Erythroid Cells (BD Bioscience, Franklin Lakes, NJ, USA). The percentage of PS exposure was measured using Novocyte flow cytometer (Agilent, Santa Clara, CA, USA). Data from 10,000 events were collected and analyzed using NovoExpress software (ver. 1.6.3) (Agilent, Santa Clara, CA, USA).

### 4.6. Ex Vivo RBC Morphology Analysis by Scanning Electron Microscopy

For morphological evaluation, blood was collected from rats injected intravenously with Ad5 vector-rAd/HA(PR8) (10^9^ OPU/rat) after incubation periods of 0.5 h, 2 h, and 4 h. RBCs were fixed in 2% glutaraldehyde, post-fixed in 1% osmium tetroxide, dehydrated through a graded ethanol series (30–100%), gold-coated, and imaged using a Merlin Compact FE-SEM (Carl Zeiss, Jena, Germany).

### 4.7. In Vivo Venous Thrombosis Model

In vivo venous thrombosis experiments were performed using rats intravenously injected with Ad5 vector-rAd/HA(PR8) at concentrations of 10^8^ or 10^9^ OPU/rat. Two hours post-injection, thrombus formation was induced in the inferior vena cava (IVC) by localized infusion of thromboplastin (RecombiPlasTin 2 G; Instrumentation Laboratory, Bedford, MA, USA) for 1 min. Blood stasis was maintained by clamping the IVC for an additional 15 min. Formed thrombi were excised from IVC segments and either weighed directly or photographed for thrombus area analysis. Thrombus area was quantified using ImageJ software (ver. 1.54) from images captured with a Stemi 305 stereomicroscope (Carl Zeiss, Jena, Germany).

### 4.8. Statistical Analysis

All statistical analyses were performed using GraphPad Prism 10 (GraphPad Software, San Diego, CA, USA). For multiple-group comparisons, data were analyzed by one-way ANOVA followed by Dunnett’s post hoc test. Comparisons between two groups were performed using a unpaired two-tailed *t*-test. The *p*-values were indicated only when statistical significance was observed. Statistical significance was defined as *p* < 0.05, and the data were presented as mean ± SEM. *p* values are reported in the figure legends when statistical significance is observed.

## Data Availability

All data supporting the findings of this study are included in the article. Additional information is available from the corresponding author (H.Y.C.) upon reasonable request.

## References

[1] Kyriakidis N.C., Lopez-Cortes A., Gonzalez E.V., Grimaldos A.B., Prado E.O. (2021). SARS-CoV-2 vaccines strategies: A comprehensive review of phase 3 candidates. NPJ Vaccines.

[2] Zhu F.C., Li Y.H., Guan X.H., Hou L.H., Wang W.J., Li J.X., Wu S.P., Wang B.S., Wang Z., Wang L. (2020). Safety, tolerability, and immunogenicity of a recombinant adenovirus type-5 vectored COVID-19 vaccine: A dose-escalation, open-label, non-randomised, first-in-human trial. Lancet.

[3] Greinacher A., Thiele T., Warkentin T.E., Weisser K., Kyrle P.A., Eichinger S. (2021). Thrombotic Thrombocytopenia after ChAdOx1 nCov-19 Vaccination. N. Engl. J. Med..

[4] Rodriguez E.V.C., Bouazza F.Z., Dauby N., Mullier F., d’Otreppe S., Jissendi Tchofo P., Bartiaux M., Sirjacques C., Roman A., Hermans C. (2022). Fatal vaccine-induced immune thrombotic thrombocytopenia (VITT) post Ad26.COV2.S: First documented case outside US. Infection.

[5] Pottegard A., Lund L.C., Karlstad O., Dahl J., Andersen M., Hallas J., Lidegaard O., Tapia G., Gulseth H.L., Ruiz P.L. (2021). Arterial events, venous thromboembolism, thrombocytopenia, and bleeding after vaccination with Oxford-AstraZeneca ChAdOx1-S in Denmark and Norway: Population based cohort study. BMJ.

[6] Dag Berild J., Bergstad Larsen V., Myrup Thiesson E., Lehtonen T., Grosland M., Helgeland J., Wolhlfahrt J., Vinslov Hansen J., Palmu A.A., Hviid A. (2022). Analysis of Thromboembolic and Thrombocytopenic Events After the AZD1222, BNT162b2, and MRNA-1273 COVID-19 Vaccines in 3 Nordic Countries. JAMA Netw. Open.

[7] Carlisle R.C., Di Y., Cerny A.M., Sonnen A.F., Sim R.B., Green N.K., Subr V., Ulbrich K., Gilbert R.J., Fisher K.D. (2009). Human erythrocytes bind and inactivate type 5 adenovirus by presenting Coxsackie virus-adenovirus receptor and complement receptor 1. Blood.

[8] Alba R., Bradshaw A.C., Parker A.L., Bhella D., Waddington S.N., Nicklin S.A., van Rooijen N., Custers J., Goudsmit J., Barouch D.H. (2009). Identification of coagulation factor (F)X binding sites on the adenovirus serotype 5 hexon: Effect of mutagenesis on FX interactions and gene transfer. Blood.

[9] Baker A.T., Boyd R.J., Sarkar D., Teijeira-Crespo A., Chan C.K., Bates E., Waraich K., Vant J., Wilson E., Truong C.D. (2021). ChAdOx1 interacts with CAR and PF4 with implications for thrombosis with thrombocytopenia syndrome. Sci. Adv..

[10] Voysey M., Clemens S.A.C., Madhi S.A., Weckx L.Y., Folegatti P.M., Aley P.K., Angus B., Baillie V.L., Barnabas S.L., Bhorat Q.E. (2021). Safety and efficacy of the ChAdOx1 nCoV-19 vaccine (AZD1222) against SARS-CoV-2: An interim analysis of four randomised controlled trials in Brazil, South Africa, and the UK. Lancet.

[11] Ayoubkhani D., Bermingham C., Pouwels K.B., Glickman M., Nafilyan V., Zaccardi F., Khunti K., Alwan N.A., Walker A.S. (2022). Trajectory of long covid symptoms after COVID-19 vaccination: Community based cohort study. BMJ.

[12] Catala M., Mercade-Besora N., Kolde R., Trinh N.T.H., Roel E., Burn E., Rathod-Mistry T., Kostka K., Man W.Y., Delmestri A. (2024). The effectiveness of COVID-19 vaccines to prevent long COVID symptoms: Staggered cohort study of data from the UK, Spain, and Estonia. Lancet Respir. Med..

[13] Krishna B.A., Metaxaki M., Wills M.R., Sithole N. (2023). Reduced Incidence of Long Coronavirus Disease Referrals to the Cambridge University Teaching Hospital Long Coronavirus Disease Clinic. Clin. Infect. Dis..

[14] Weisel J.W., Litvinov R.I. (2019). Red blood cells: The forgotten player in hemostasis and thrombosis. J. Thromb. Haemost..

[15] Alkarithi G., Duval C., Shi Y., Macrae F.L., Ariens R.A.S. (2021). Thrombus Structural Composition in Cardiovascular Disease. Arter. Thromb. Vasc. Biol..

[16] Andrews N.J., Stowe J., Ramsay M.E., Miller E. (2022). Risk of venous thrombotic events and thrombocytopenia in sequential time periods after ChAdOx1 and BNT162b2 COVID-19 vaccines: A national cohort study in England. Lancet Reg. Health Eur..

[17] Weisel J.W., Litvinov R.I. (2021). Visualizing thrombosis to improve thrombus resolution. Res. Pract. Thromb. Haemost..

[18] Whelihan M.F., Zachary V., Orfeo T., Mann K.G. (2012). Prothrombin activation in blood coagulation: The erythrocyte contribution to thrombin generation. Blood.

[19] Chernysh I.N., Nagaswami C., Kosolapova S., Peshkova A.D., Cuker A., Cines D.B., Cambor C.L., Litvinov R.I., Weisel J.W. (2020). The distinctive structure and composition of arterial and venous thrombi and pulmonary emboli. Sci. Rep..

[20] Tutwiler V., Mukhitov A.R., Peshkova A.D., Le Minh G., Khismatullin R.R., Vicksman J., Nagaswami C., Litvinov R.I., Weisel J.W. (2018). Shape changes of erythrocytes during blood clot contraction and the structure of polyhedrocytes. Sci. Rep..

[21] Lyons M., Onion D., Green N.K., Aslan K., Rajaratnam R., Bazan-Peregrino M., Phipps S., Hale S., Mautner V., Seymour L.W. (2006). Adenovirus type 5 interactions with human blood cells may compromise systemic delivery. Mol. Ther..

[22] Sun S., Campello E., Zou J., Konings J., Huskens D., Wan J., Fernandez D.I., Reutelingsperger C.P.M., Ten Cate H., Toffanin S. (2023). Crucial roles of red blood cells and platelets in whole blood thrombin generation. Blood Adv..

[23] Thomas T., Stefanoni D., Dzieciatkowska M., Issaian A., Nemkov T., Hill R.C., Francis R.O., Hudson K.E., Buehler P.W., Zimring J.C. (2020). Evidence of Structural Protein Damage and Membrane Lipid Remodeling in Red Blood Cells from COVID-19 Patients. J. Proteome Res..

[24] Eder J., Schumm L., Armann J.P., Puhan M.A., Beuschlein F., Kirschbaum C., Berner R., Toepfner N. (2023). Increased red blood cell deformation in children and adolescents after SARS-CoV-2 infection. Sci. Rep..

[25] Kim J.Y., Choi Y., Nguyen H.H., Song M.K., Chang J. (2013). Mucosal immunization with recombinant adenovirus encoding soluble globular head of hemagglutinin protects mice against lethal influenza virus infection. Immune Netw..

[26] Folegatti P.M., Ewer K.J., Aley P.K., Angus B., Becker S., Belij-Rammerstorfer S., Bellamy D., Bibi S., Bittaye M., Clutterbuck E.A. (2020). Safety and immunogenicity of the ChAdOx1 nCoV-19 vaccine against SARS-CoV-2: A preliminary report of a phase 1/2, single-blind, randomised controlled trial. Lancet.

[27] Sadoff J., Le Gars M., Shukarev G., Heerwegh D., Truyers C., de Groot A.M., Stoop J., Tete S., Van Damme W., Leroux-Roels I. (2021). Interim Results of a Phase 1-2a Trial of Ad26.COV2.S Covid-19 Vaccine. N. Engl. J. Med..

[28] Semeraro F., Ammollo C.T., Esmon N.L., Esmon C.T. (2014). Histones induce phosphatidylserine exposure and a procoagulant phenotype in human red blood cells. J. Thromb. Haemost..

[29] Yan M., Xu M., Li Z., An Y., Wang Z., Li S., Chen Y., Xia Y., Wang L., Wang L. (2022). TMEM16F mediated phosphatidylserine exposure and microparticle release on erythrocyte contribute to hypercoagulable state in hyperuricemia. Blood Cells Mol. Dis..

[30] Owens A.P., Mackman N. (2010). Tissue factor and thrombosis: The clot starts here. Thromb. Haemost..

[31] Wang J., Yu C., Zhuang J., Qi W., Jiang J., Liu X., Zhao W., Cao Y., Wu H., Qi J. (2022). The role of phosphatidylserine on the membrane in immunity and blood coagulation. Biomark. Res..

[32] Cines D.B., Greinacher A. (2023). Vaccine-induced immune thrombotic thrombocytopenia. Blood.

[33] Chung H.Y., Bian Y., Lim K.M., Kim B.S., Choi S.H. (2022). MARTX toxin of Vibrio vulnificus induces RBC phosphatidylserine exposure that can contribute to thrombosis. Nat. Commun..

[34] Bouazzaoui A., Abdellatif A.A.H. (2024). Vaccine delivery systems and administration routes: Advanced biotechnological techniques to improve the immunization efficacy. Vaccine X.

[35] Kudlay D., Svistunov A., Satyshev O. (2022). COVID-19 Vaccines: An Updated Overview of Different Platforms. Bioengineering.

[36] Stebbings R., Armour G., Pettis V., Goodman J. (2022). AZD1222 (ChAdOx1 nCov-19): A Single-Dose biodistribution study in mice. Vaccine.

[37] Azzarone B., Veneziani I., Moretta L., Maggi E. (2021). Pathogenic Mechanisms of Vaccine-Induced Immune Thrombotic Thrombocytopenia in People Receiving Anti-COVID-19 Adenoviral-Based Vaccines: A Proposal. Front. Immunol..

[38] Wang P., Zheng L., Yan S., Xuan X., Yang Y., Qi X., Dong H. (2024). Understanding the role of red blood cells in venous thromboembolism: A comprehensive review. Am. J. Med. Sci..

[39] Zanetto A., Campello E., Bulato C., Willems R., Konings J., Roest M., Gavasso S., Nuozzi G., Toffanin S., Zanaga P. (2024). Whole blood thrombin generation shows a significant hypocoagulable state in patients with decompensated cirrhosis. J. Thromb. Haemost..

[40] Willems R.A.L., Konings J., Huskens D., Middelveld H., Pepels-Aarts N., Verbeet L., de Groot P.G., Heemskerk J.W.M., Ten Cate H., de Vos-Geelen J. (2024). Altered whole blood thrombin generation and hyperresponsive platelets in patients with pancreatic cancer. J. Thromb. Haemost..

[41] Peshkova A.D., Rednikova E.K., Khismatullin R.R., Kim O.V., Muzykantov V.R., Purohit P.K., Litvinov R.I., Weisel J.W. (2025). Red blood cell aggregation within a blood clot causes platelet-independent clot shrinkage. Blood Adv..

[42] Pavord S., Scully M., Hunt B.J., Lester W., Bagot C., Craven B., Rampotas A., Ambler G., Makris M. (2021). Clinical Features of Vaccine-Induced Immune Thrombocytopenia and Thrombosis. N. Engl. J. Med..

[43] Perry R.J., Tamborska A., Singh B., Craven B., Marigold R., Arthur-Farraj P., Yeo J.M., Zhang L., Hassan-Smith G., Jones M. (2021). Cerebral venous thrombosis after vaccination against COVID-19 in the UK: A multicentre cohort study. Lancet.

[44] Cho J., Jo H., Kim H., Park J., Pizzol D., Kim M.S., Woo H.G., Yon D.K. (2025). Global Burden of Vaccine-Associated Cerebrovascular Venous Sinus Thrombosis, 1968–2024: A Critical Analysis From the WHO Global Pharmacovigilance Database. J. Korean Med. Sci..

[45] Mudrick H.E., Lu S.C., Bhandari J., Barry M.E., Hemsath J.R., Andres F.G.M., Ma O.X., Barry M.A., Reddy V.S. (2024). Structure-derived insights from blood factors binding to the surfaces of different adenoviruses. Nat. Commun..

[46] Caspary L., Shaw J.R., Stalder O., Brodard J., Angelillo-Scherrer A., Vrotniakaite-Bajerciene K. (2025). Clinical utility of thrombin generation using ST-Genesia(R) in patients with hereditary and acquired thrombophilia: A cross-sectional study. Thromb. Res..

[47] Zanetto A., Campello E., Bulato C., Willems R., Konings J., Roest M., Gavasso S., Nuozzi G., Toffanin S., Burra P. (2025). Impaired whole blood thrombin generation is associated with procedure-related bleeding in acutely decompensated cirrhosis. J. Hepatol..

[48] Ma J., Duffy M.R., Deng L., Dakin R.S., Uil T., Custers J., Kelly S.M., McVey J.H., Nicklin S.A., Baker A.H. (2015). Manipulating adenovirus hexon hypervariable loops dictates immune neutralisation and coagulation factor X-dependent cell interaction in vitro and in vivo. PLoS Pathog..

[49] Bliss C.M., Hulin-Curtis S.L., Williams M., Maruskova M., Davies J.A., Statkute E., Baker A.T., Stack L., Kerstetter L., Kerr-Jones L.E. (2024). A pseudotyped adenovirus serotype 5 vector with serotype 49 fiber knob is an effective vector for vaccine and gene therapy applications. Mol. Ther. Methods Clin. Dev..

[50] Sadoff J., Gray G., Vandebosch A., Cardenas V., Shukarev G., Grinsztejn B., Goepfert P.A., Truyers C., Van Dromme I., Spiessens B. (2022). Final Analysis of Efficacy and Safety of Single-Dose Ad26.COV2.S. N. Engl. J. Med..

[51] Zhu Y., Tang R., Li X., Chen X., Wang X., Wang Y., Wang R., Zhu F., Li J. (2024). Vaccination with Adenovirus Type 5 Vector-Based COVID-19 Vaccine as the Primary Series in Adults: A Randomized, Double-Blind, Placebo-Controlled Phase 1/2 Clinical Trial. Vaccines.

